# Accuracy of ChatGPT-3.5, ChatGPT-4o, Copilot, Gemini, Claude, and Perplexity in advising on lumbosacral radicular pain against clinical practice guidelines: cross-sectional study

**DOI:** 10.3389/fdgth.2025.1574287

**Published:** 2025-06-27

**Authors:** Giacomo Rossettini, Silvia Bargeri, Chad Cook, Stefania Guida, Alvisa Palese, Lia Rodeghiero, Paolo Pillastrini, Andrea Turolla, Greta Castellini, Silvia Gianola

**Affiliations:** ^1^ School of Physiotherapy, University of Verona, Verona, Italy; ^2^ Department of Physiotherapy, Faculty of Medicine, Health and Sports, Universidad Europea de Madrid, Madrid, Spain; ^3^ Unit of Clinical Epidemiology, IRCCS Istituto Ortopedico Galeazzi, Milan, Italy; ^4^ Department of Orthopaedics, Duke University, Durham, NC, United States; ^5^ Duke Clinical Research Institute, Duke University, Durham, NC, United States; ^6^ Department of Population Health Sciences, Duke University, Durham, NC, United States; ^7^ Department of Medical Sciences, University of Udine, Udine, Italy; ^8^ Department of Rehabilitation, Hospital of Merano (SABES-ASDAA), Teaching Hospital of Paracelsus Medical University (PMU), Merano-Meran, Italy; ^9^ Department of Biomedical and Neuromotor Sciences (DIBINEM), Alma Mater University of Bologna, Bologna, Italy; ^10^ Unit of Occupational Medicine, IRCCS Azienda Ospedaliero-Universitaria di Bologna, Bologna, Italy

**Keywords:** artificial intelligence, physiotherapy, machine learning, musculoskeletal, natural language processing, orthopaedics, ChatGPT, chatbots

## Abstract

**Introduction:**

Artificial Intelligence (AI) chatbots, which generate human-like responses based on extensive data, are becoming important tools in healthcare by providing information on health conditions, treatments, and preventive measures, acting as virtual assistants. However, their performance in aligning with clinical practice guidelines (CPGs) for providing answers to complex clinical questions on lumbosacral radicular pain is still unclear. We aim to evaluate AI chatbots' performance against CPG recommendations for diagnosing and treating lumbosacral radicular pain.

**Methods:**

We performed a cross-sectional study to assess AI chatbots' responses against CPGs recommendations for diagnosing and treating lumbosacral radicular pain. Clinical questions based on these CPGs were posed to the latest versions (updated in 2024) of six AI chatbots: ChatGPT-3.5, ChatGPT-4o, Microsoft Copilot, Google Gemini, Claude, and Perplexity. The chatbots' responses were evaluated for (a) consistency of text responses using Plagiarism Checker X, (b) intra- and inter-rater reliability using Fleiss' Kappa, and (c) match rate with CPGs. Statistical analyses were performed with STATA/MP 16.1.

**Results:**

We found high variability in the text consistency of AI chatbot responses (median range 26%–68%). Intra-rater reliability ranged from “almost perfect” to “substantial,” while inter-rater reliability varied from “almost perfect” to “moderate.” Perplexity had the highest match rate at 67%, followed by Google Gemini at 63%, and Microsoft Copilot at 44%. ChatGPT-3.5, ChatGPT-4o, and Claude showed the lowest performance, each with a 33% match rate.

**Conclusions:**

Despite the variability in internal consistency and good intra- and inter-rater reliability, the AI Chatbots' recommendations often did not align with CPGs recommendations for diagnosing and treating lumbosacral radicular pain. Clinicians and patients should exercise caution when relying on these AI models, since one to two-thirds of the recommendations provided may be inappropriate or misleading according to specific chatbots.

## Introduction

Large Language Models (LLMs) are deep learning systems capable of producing, understanding, and interacting with human language (1). In the field of LLMs, artificial intelligence (AI) chatbots (e.g., ChatGPT, Google Gemini, Microsoft Copilot) represent emerging tools that use algorithms to predict and generate words and phrases based on provided text input (2, 3). Recently, notable hype involving AI Chatbots has occurred because of their friendly interface that facilitates interaction, thus simplifying user accessibility (4).

This progress is relevant in health care, where patients increasingly use AI chatbots to navigate health-related queries (5). AI chatbots allow patients to inquire about their health conditions, treatment options, and preventive measures by acting as virtual assistants (6). However, the risk of misinformation, prejudices, lack of transparency, and hesitations about privacy and data security are still unresolved issues (7, 8). The AI Chatbots' ability to facilitate patient health literacy underlines the importance of investigating their performance in providing health information, guaranteeing reliability, accuracy, and alignment of their content with the best evidence included in the clinical practice guidelines (CPGs) (9).

Focusing on musculoskeletal pain conditions of the lumbar spine, conflicting evidence emerged when assessing the performance of AI Chatbots agreement with CPGs (10–13). A comparative analysis of ChatGPT's responses to CPGs for degenerative spondylolisthesis revealed a concordance rate of 46.4% for ChatGPT-3.5, while 67.9% for ChatGPT-4 (10). Another study reported a ChatGPT-3.5 accuracy of 65% in generating clinical recommendations for low back pain, which improved to 72% when prompted by an experienced orthopaedic surgeon (12). While assessing recommendations regarding lumbar disk herniation with radiculopathy, Mejia et al. found that ChatGPT-3.5 and ChatGPT-4 provided matched accuracy when compared to the CPGs of 52% and 59% of responses, respectively (11). Recently, ChatGPT-3.5 showed limited word text consistency of responses in terms of low levels of agreement between different parts of a system and percentage match rate with CPGs for lumbosacral radicular pain, presenting agreement of responses (i.e., match rate) in only 33% of recommendations (13).

Despite growing interest in the use of AI chatbots to support patient education in musculoskeletal conditions, current evidence reveals substantial variability in their response accuracy when compared with established CPGs (10–13). Most existing studies have evaluated individual chatbots or outdated versions (e.g., ChatGPT 3.5), without direct comparisons across multiple and updated models (e.g., ChatGPT-4o). Moreover, limited data are available on the performance of newer AI systems, such as Google Gemini, Microsoft Copilot, Claude, and Perplexity, when benchmarked against consistent, evidence-based recommendations for specific conditions like lumbosacral radicular pain. This lack of comprehensive, up-to-date comparison represents a critical gap in the literature, particularly regarding the reliability and accuracy (i.e., match rate) of the information these tools provide in the context of musculoskeletal care (4).

Therefore, the aim of this study was to compare the performance of five emerging AI Chatbots (ChatGPT-4o, Google Gemini, Microsoft Copilot, Claude, and Perplexity) and ChatGPT-3.5 (13) in providing accurate, evidence-based health advice for lumbosacral radicular pain against CPGs. In detail, we assessed (a) the word text consistency of chatbots, (b) intra- and inter-rater reliability of readers, and (c) match rate of each AI Chatbots with CPG recommendations.

## Materials and methods

### Study design and ethics

**Figure 1:**
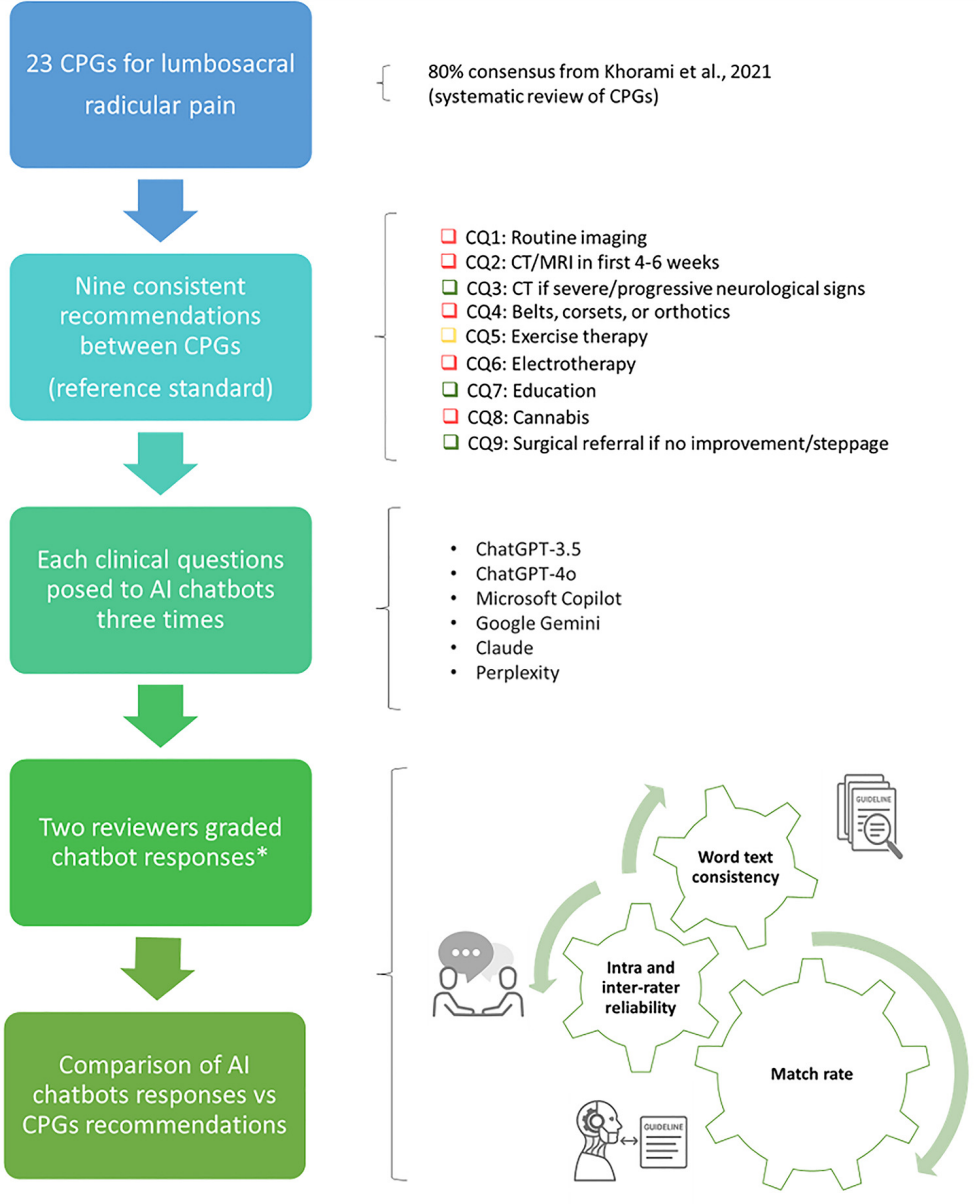
Overview of the study's pipeline. AI, artificial intelligence; CPGs, clinical practice guidelines; CQ, clinical question. *According to criteria for clinical inference adopted by the CPGs review appraisal (14). Green: “should do” (i.e., strong recommendation in favour to intervention); yellow: “could do” (i.e., weak recommendation in favour to intervention); red: “do not do” (i.e., recommendation against intervention); blue: “uncertain” (i.e., direction and strength of recommendation unclear).

We performed an observational cross-sectional study, comparing the recommendations of a systematic review of CPGs (14) with those of AI Chatbots for lumbosacral radicular pain (Figure 1). We followed the Strengthening the Reporting of Observational Studies in Epidemiology guideline (STROBE) (15) and Reporting guideline for the early-stage clinical evaluation of decision support systems driven by artificial intelligence (DECIDE-AI) to achieve high-quality standards for reporting (16). As the units in our investigation were studies and not participants, we did not involve any interaction with human subjects or access to identifiable private information: ethical approval was not considered necessary (17).

### Setting

In April 2024, a multidisciplinary group of methodologists, clinicians, and researchers with diverse healthcare backgrounds (e.g., physiotherapy, nursing) and expertise (e.g., musculoskeletal, neurology) coordinated this study. This choice of multiple backgrounds was aimed to ensure clinical expertise and to reflect the systematic appraisal of the recommendations of the Standards for Development of Trustworthy CPGs (18).

### Sample

In accordance with our previous study on AI Chatbots (13), we used a sample of 9 CPGs recommendations for patients with lumbosacral radicular pain that emerged from a recent systematic review (14). A typical lumbosacral radicular pain pattern radiates through the path of a spinal nerve. It usually originates from the lumbar (lower back) or sacral (lowermost part of the spine) regions and may spread to the buttocks, thighs, or legs (14).

The nine CPGs recommendations included physical examination and diagnostics, non-invasive interventions, pharmacological interventions, invasive treatments and referral (14). We used these as reference standards, because their strength and direction were consistent across all CPGs for lumbosacral radicular pain (consensus ≥80% CPGs indicating “should do”, “could do”, “do not do”, or “uncertain”) (19). From the nine consistent recommendations, the multidisciplinary group developed nine relative clinical questions that were inputted into the five AI Chatbots (13) (Table 1).

**Table 1:** Clinical questions obtained from the selected consistent recommendations across multiple clinical practice guidelines (14).

Area	Clinical questions
Diagnostics	CQ1. “Should routine imaging be offered in primary care or absent of red flags in patients with low back pain and/or sciatica?”
	CQ2. “Should Computed Tomography (CT)/ Magnetic resonance imaging (MRI) be offered in first 4–6 weeks in people with low back pain and/or sciatica?”
	CQ3. “When history and physical examination findings are consistent with disc herniation, should CT be offered after 4–6 weeks of low back pain with severe or progressive neurologic signs and/or symptoms?”
Non-invasive interventions	CQ4. “Should devices (such as belts, corset, and/or foot orthotics) be used in the management of non-specific low back pain and sciatica?”
	CQ5. “Should exercises therapies be used in the management of non-specific low back pain and sciatica?”
	CQ6. “Should electrotherapies (such as TENS/PENS/interferential therapy) be used in the management of non-specific low back pain and sciatica?”
	CQ7. “Should educational care be used in the management of non-specific low back pain and sciatica?”
Pharmacological interventions	CQ8. “Should cannabis be used in the management of non-specific low back pain and sciatica?”
Invasive treatments	CQ9. “Should referral to a surgeon be done when there is no improvement of symptoms with conservative therapy, or immediately when there is steppage gait in non-specific low back pain and sciatica?”

CQ, clinical question; TENS, transcutaneous electrical nerve stimulation, PENS, percutaneous electrical nerve stimulation.

### Measurements and variables

We used the latest versions of the AI chatbots that were updated in April 2024, including: ChatGPT-4o (OpenAI Incorporated, Mission District, San Francisco, United States) (20), Microsoft Copilot (Microsoft Corporation, WA, US) (21), Google Gemini (Alphabet Inc., CA, US) (22), Claude (Anthropic PBC, San Francisco, California, U.S.) (23), and Perplexity (Perplexity AI, Inc., San Francisco, California, USA) (24). The CPGs were compared with the responses of the five emerging AI Chatbots (ChatGPT-4o, Google Gemini, Microsoft Copilot, Claude, and Perplexity) and ChatGPT-3.5 (13). We considered the following variables for the analysis: (a) word text consistency of chatbots, (b) intra- and inter-rater reliability of those reading the AI chatbots, and (c) match rate of AI chatbots (13).

Text consistency of responses represents the degree of the interrelatedness among the items (i.e., text words) following the international consensus on taxonomy, terminology, and definitions of measurement properties for health-related patient-reported outcomes (COSMIN) (25, 26). To measure the word text consistency of the AI chatbot answers, we adopted Plagiarism Checker X (27) to check for overlapping text among the three answers. The software compares the text of two documents side by side and displays the degree of similarity as a percentage (range: 0%–100%). A 0% match indicates no similarity, whereas a 100% match suggests a complete overlap of words and perfect consistency within the document. An example is given in Supplementary File 1, Figure S1.

Intra and inter-rater reliability indicate the level of agreement among independent reviewers in rating the three text responses obtained on the same clinical question (26). Match rate expresses the accuracy of how close a measurement result is to the measure's value, representing a qualitative performance characteristics (28). To measure the match rate as agreement of grading AI chatbot answers against the CPGs recommendations, we compared the frequency of the same judgements between AI chatbots and CPGs recommendations.

### Procedure

To avoid prompt engineering influencing the generative output, we standardized the input formats of the nine clinical questions following the Prompt-Engineering-Guide (29). First, we prepared the nine clinical questions in Microsoft Word® by formatting them for proper structure and readability. Then, we manually copied and pasted each question onto the five AI chatbots during a single chat session on April 1, 2024.

The clinical questions were run three times to assess word text consistency, and responses were recorded (13). To minimize learning bias and eliminate the influence of prior interactions, we: (a) created and used a new account, (b) did not provide positive or negative feedback on the answer given, and (c) deleted conversations with the AI chatbots before entering each new question into a new chat (with no previous conversations) (13). To further enhance robustness and reproducibility, we implemented a controlled input/output protocol: all prompts were delivered in isolated, single-turn sessions, and the resulting outputs were copied verbatim, anonymized, and stored offline to prevent contextual contamination or *post hoc* alteration (13).

To measure intra and inter-rater reliability, two reviewers (SB, SG) with expertise in musculoskeletal disorders and clinical epidemiology (more than 3 years) graded each set of three text responses from the AI chatbot for all clinical questions. Prior to the study, they received 5 h of training. Using the same criteria for clinical inference adopted by the CPGs review appraisal (14), reviewers graded each set of responses as follows: “should do” (i.e., strong recommendation in favour to intervention), “could do” (i.e., weak recommendation in favour to intervention), “do not do” (i.e., recommendation against intervention) or “uncertain” (i.e., direction and strength of recommendation unclear). The terminology used for the grading system can be found in Supplementary File 1, Table S1.

We compared AI Chatbots' text responses to CPGs' recommendations in answering the nine clinical questions to measure their match rate (13). To obtain an unambiguous clinical judgement on AI Chatbot answers for each reviewer, we considered the mode (i.e., how frequently a particular categorial variable occurs) of the three trials of each categorical variable. A final clinical judgement between reviewers was established. We consulted a third reviewer (ZI) if there was any disagreement.

### Statistical analyses

STATA/MP 16.1 was used to perform all statistical calculations, while data were plotted using STATA and Python. Categorical data were presented as absolute frequencies and percentages (%). A *p*-value of <.05 was considered significant. We *a priori* followed a common rule of thumb for defining word text consistency: ≥90% “excellent”, 80%–90% “good”, 70%–80% “acceptable”, 60%–70% “questionable”, 50%–60% “poor”, and <50% “unacceptable” (30). For intra and inter-rater reliability on AI Chatbot answers, we adopted Fleiss' Kappa (*κ*) (31). Interpretation of strength of agreement (Kappa-values) was categorized following Landis and Koch suggestions (<0.00 “poor”, 0–0.20 “slight”; 0.21–0.40 “fair”, 0.41–0.60 “moderate”, 0.61–0.80 “substantial”, 0.81–1.00 “almost perfect”) (32). As a measure of match rate, we used the inter-observer agreement obtained from a formula that divides the number of agreements in the grading by the sum of the agreement and disagreement [No. of agreements/(No. of agreements + disagreements) × 100]. A chi-square test was used to ascertain whether the answers differed among all AI chatbots against the CPGs' recommendations. A *p*-value of <.05 was considered significant. Since we compared six groups/AI chatbots, a Bonferroni adjustment for multiple measures was applied. Raw data are reported in Open Science Framework (OSF) repository available at https://osf.io/8dgrx/.

### Ethics

Ethical approval is not applicable as no patients were recruited or involved in this study.

## Results

### Word text consistency of AI Chatbot answers

The consistency of text responses for each Chatbot in every CQ is highly variable ranging from “unacceptable” (median 26%) to “questionable” (median 68%). Findings for each clinical question are reported in Supplementary File 2, Tables S1–S6.

### Reliability of AI Chatbot answers

The intra-rater reliability was “almost perfect” for both reviewers considering Microsoft Copilot, Perplexity and ChatGPT-3.5 and “substantial” for ChatGPT-4o, Cloud and Gemini. Out of nine CQ ratings, the inter-rater reliability between the two reviewers was “almost perfect” for Perplexity (0.84, SE: 0.16) and ChatGPT-3.5 (0.85, SE: 0.15), “substantial” for Microsoft Copilot (0.69, SE: 0.20), Cloude (0.66, SE: 0.21) and Google Gemini (0.80, SE: 0.18), and “moderate” for ChatGPT-4o (0.54, SE: 0.23). Table 2 reported the Kappa Fleiss for each chatbot.

**Table 2:** Intra and inter-rater reliability of AI chatbot answer.

AI chatbots	Reviewer 1	Reviewer 2	Reviewer 1 vs. Reviewer 2
	*K* (SE)	*K* (SE)	*K* (SE)
ChatGPT-3.5^a^	0.90 (0.09)	0.90 (0.10)	0.85 (0.15)
ChatGPT-4O	0.79 (0.14)	0.70 (0.15)	0.54 (0.23)
Cloude	0.79 (0.13)	0.75 (0.17)	0.66 (0.21)
Microsoft Copilot	1.0 (0)	0.89 (0.11)	0.69 (0.20)
Google Gemini	0.76 (0.16)	0.74 (0.16)	0.80 (0.18)
Perplexity	0.89 (0.11)	0.90 (0.1)	0.84 (0.16)

K, Kappa Fleiss; SE, standard error.

^a^ Data from Gianola et al. (13).

### Match rate of AI Chatbot answers compared to CPGs recommendations

**Figure 2:**
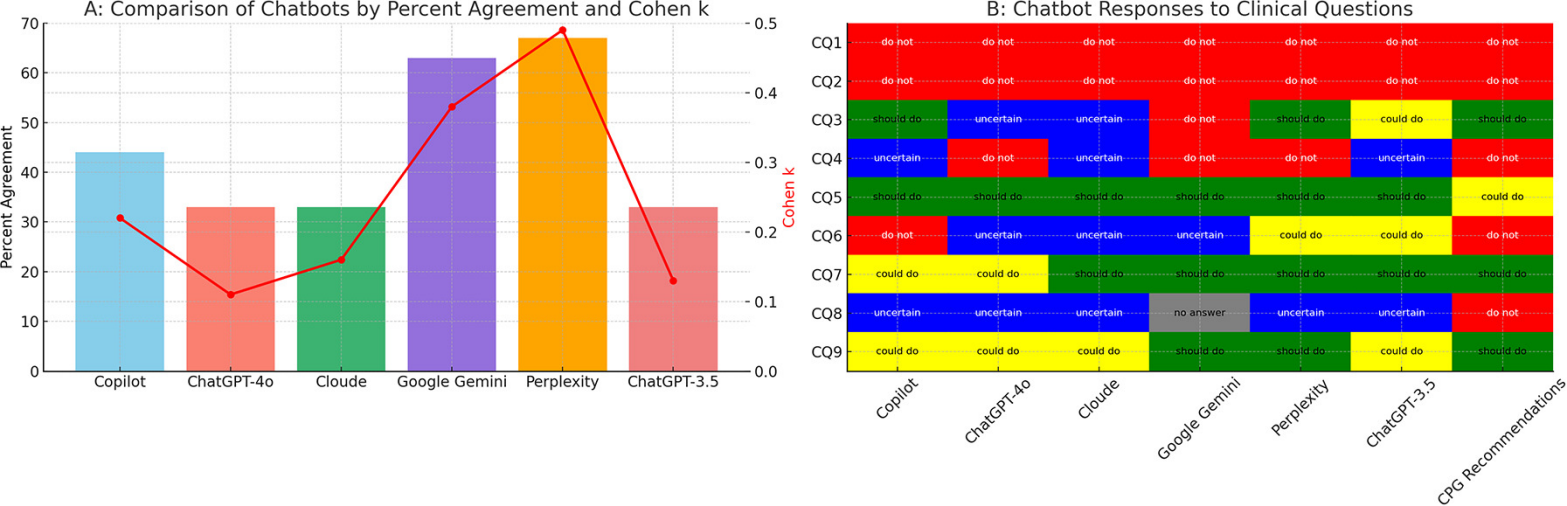
Performance of AI chatbots compared to CPG recommendation. AI, artificial intelligence; CPGs, clinical practice guidelines; CQ, clinical question. **(A)** Quantitative bar chart representing percentage of agreement (*y*-axis, left side) of the six chatbots (*x*-axis). Red points represent Cohen's *K* value (*y*-axis, right side). **(B)** Qualitative table showing the clinical inference for each CQ (*y*-axis) in each chatbot (*x*-axis). Chat GPT-3.5 data are from Gianola et al. (13).

Among the AI Chatbots evaluated, Perplexity exhibited the highest matched rate at 67%, followed by Google Gemini at 63% and Microsoft Copilot at 44%. Conversely, Cloude, ChatGPT-3.5, and ChatGPT-4o demonstrated the lowest match rates with a score of 33% (Figure 2, Table 3).

**Table 3:** Inter-observer agreement (IOA).

CQ	ChatGPT-3.5^a^	ChatGPT-4o	Cloude	Microsoft Copilot	Google Gemini	Perplexity	CPGs
CQ1	Do not	Do not	Do not	Do not	Do not	Do not	Do not
CQ2	Do not	Do not	Do not	Do not	Do not	Do not	Do not
CQ3	Could do	Uncertain	Uncertain	Should do	Do not	Should do	Should do
CQ4	Uncertain	Do not	Uncertain	Uncertain	Do not	Do not	Do not
CQ5	Should do	Should do	Should do	Should do	Should do	Should do	Could do
CQ6	Could do	Uncertain	Uncertain	Do not	Uncertain	Could do	Do not
CQ7	Should do	Could do	Should do	Could do	Should do	Should do	Should do
CQ8	Uncertain	Uncertain	Uncertain	Uncertain	No answer	Uncertain	Do not
CQ9	Could do	Could do	Could do	Could do	Should do	Should do	Should do
Match rate	33%	33%	33%	44%	63%	67%	-
Cohen (SD)	0.13 (0.16)	0.11 (0.12)	0.16 (0.14)	0.22 (0.17)	0.38 (0.25)	0.49 (0.20)	-

CPG, clinical practice guideline; CQ, clinical questions; %, percentage; SD, standard deviation.

^a^ Data from Gianola et al. (13).

## Discussion

### Main findings

In this study, we compared the performance of five updated AI Chatbots (ChatGPT-4o, Google Gemini, Microsoft Copilot, Claude, and Perplexity) and ChatGPT-3.5 (13) in producing evidence-based health advice against CPGs for radicular lumbosacral pain. As the main finding, no AI chatbots provided advice that was in absolute agreement with CPGs, confirming the results previously found in other lumbar spine pain conditions (10–13). This finding suggests that although AI chatbots have promising potential, they currently do not perform adequately to be recommended for patient use.

### Comparison with evidence

Comparing our study with existing literature is a challenge due to the limited amount of research that has examined multiple AI chatbots (e.g., mainly ChatGPT-3.5 and 4) against CPGs (10–13) and that have analysed similar performance metrics (13). We observed that: (a) the word consistency of text responses for each Chatbot was highly variable; (b) the intra-rater reliability ranged from “almost perfect” to “substantial”, whereas the inter-rater reliability varied from “almost perfect” to “moderate”; and (c) the match rate differs notably between AI Chatbots, with Gemini and Perplexity being superior, albeit imperfect.

The findings reveal substantial variability in AI chatbot performance, which likely arises from fundamental differences in model architecture (e.g., decoder-only transformer frameworks), pre-training strategies (e.g., autoregressive language modeling vs. instruction tuning), and the nature of training datasets—often heterogeneous, non-curated, and lacking peer-reviewed medical content (33). Moreover, current LLMs do not exhibit structured clinical reasoning but operate through probabilistic pattern matching, which contributes to recurrent errors such as factual inaccuracies, overgeneralization, contextual misinterpretation, and poor management of clinical ambiguity (34). This is further compounded by the lack of transparency surrounding proprietary algorithms, rendering these models “black boxes” with limited interpretability for the scientific community (11).

### Implications for clinical practice

Our results discourage the adoption of AI Chatbots as an information tool for patients with lumbosacral radicular pain. Our experience supports previously documented evidence that AI chatbots tend to provide generic, verbose, incomplete, outdated, or inaccurate information (7, 8). Furthermore, being highly dependent on the quality of the training data, AI Chatbots may be biased (e.g., language, gender, race), which affects their outputs (7, 8). Finally, AI Chatbots suffer from the phenomenon of “artificial hallucination” and may produce confident answers based on fabricated facts without evidence (7, 8).

In an era of digitisation, where patients increasingly search for health information on the web and assume it is reliable and valid (35), AI Chatbots, being user-friendly (5) have the potential to complement existing web tools (e.g., Dr Google, Wikipedia) (36, 37). However, lacking critical analysis and abstract reasoning, as well as the clinician's experience and judgement, AI Chatbots may play the role of threats rather than opportunities (4). For example, in lumbosacral radicular pain, basing their outputs on information retrieved on the web whose quality is poor (38), they could act as a nocebo source capable of spreading negative information and perpetuating an infodemic (39). As a consequence, patients with lumbosacral radicular pain could be harmed, directed towards non-evidence-based treatments, and wasted economic resources (4).

For AI chatbots to be gradually integrated into healthcare systems, clinicians, healthcare organisations, and policy-makers should raise awareness among stakeholders (e.g., patients and laypersons) with public information campaigns to analyse the pros and cons (40). It is essential that AI chatbots are trained to search for information in healthcare databases (e.g., PubMed, Scopus, Web of Science) and assess the methodological quality of the information obtained (10, 11). Accordingly, several healthcare-specific LLMs are being developed (e.g., PMC-LLaMA, Med-LLaMA, Almanac) (41–43), but further studies are needed to assess their feasibility and validity, prior to adoption.

In this evolving context, universities and academic institutions have a crucial role in both managing risks and supporting the responsible use of AI chatbots in healthcare (44). As centers of education and research, they should integrate digital health literacy into healthcare training programs, equipping future clinicians with the skills needed to critically evaluate AI tools (45). Furthermore, through interdisciplinary collaborations, universities can lead the rigorous validation of AI systems and promote the development of ethical and safe digital solutions.

### Strengths and limitations

This study is the first to compare the performance of multiple AI Chatbots against CPGs for lumbosacral radicular pain, adopting a transparent methodology that comprises the use of standardized prompts and an objective measure of performance (13). Despite this, there are some limitations that exist. Firstly, we studied five AI Chatbots that although very popular, do not represent the totality of available tools [e.g., DeepSeek (46)]. Secondly, not all the Chatbots considered were available without payment: only five were free (13, 21–24), whereas ChatGPT-4o was available at a fee (20). Given the evolving nature of LLMs, our results may not be extendable to more recent models, as AI Chatbots are continuously developed and improved (12). Thirdly, we investigated the performance of AI chatbots focusing on lumbosacral radicular pain; however, these findings may not be generalisable to other pathologies of the lumbar spine (10–13). Lastly, we did not execute a sentiment analysis on the AI's outputs to identify the quality of the texts' emotional tone (e.g., positive, negative, or neutral) (4).

Thus, while awaiting shared reporting guidelines (47), further research is needed to address the current limitations. This includes evaluating emerging and updated AI chatbot models against CPGs in other musculoskeletal conditions (e.g., upper and lower limbs, cervical and thoracic spine), assessing their interpretability, and examining performance when interpreted by end-users such as patients. Comparative studies involving human clinicians as reference standards are also warranted to determine the clinical utility of these tools. Finally, future work should explore multimodal outputs (e.g., text, visuals) and the integration of AI chatbots into patient-facing digital health platforms to enhance usability and relevance in real-world settings.

## Conclusion

In our study, none of the AI chatbots fully matched responses of the CPGs for lumbosacral radicular pain, revealing a high variability in their performance. These findings confirm that currently patients without clinician supervision cannot use AI Chatbots to provide health information.

## Data Availability

The datasets presented in this study can be found in online repositories. The names of the repository/repositories and accession number(s) can be found below: https://osf.io/8dgrx/.
