# Supplementary material for: Accuracy of ChatGPT-3.5, ChatGPT-4o, Copilot, Gemini, Claude, and Perplexity in advising on lumbosacral radicular pain against clinical practice guidelines: cross-sectional study
Source: Front Digit Health. 2025 Jun 27;7:1574287. doi: 10.3389/fdgth.2025.1574287 (PMC12245906; doi:10.3389/fdgth.2025.1574287)
Supplement: Supplementary file 1 [file Table1.docx]

**Supplementary material**

# **Supplementary File 1. eMethods**

## **Internal consistency**

### **Figure S1.** Example of Plagiarism Checker X on clinical question 1, trial 1 and 2 (ChatGPT 4o)


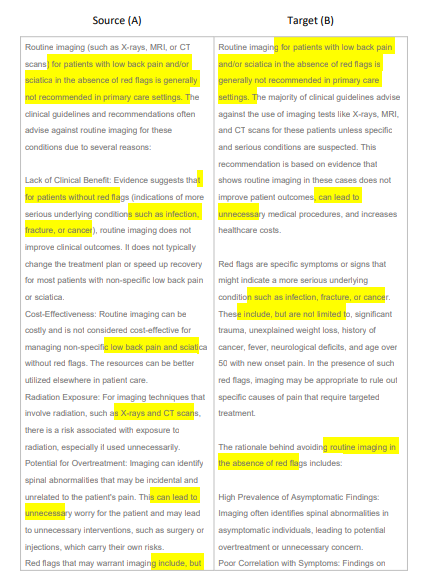

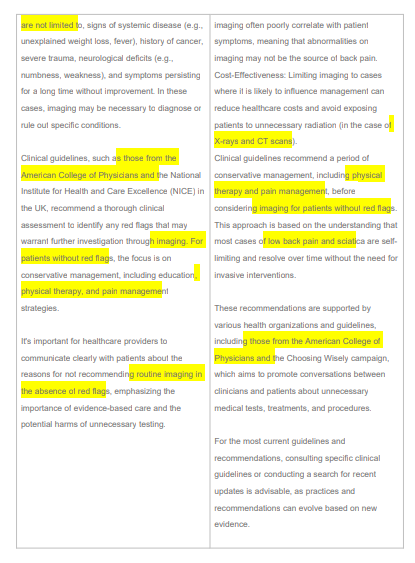


### **Table S1.** Terminology used in guidelines

| **Clinical Inference of recommendations** | **Definition** | **Examples of Terminology Used in**  **Guidelines** |
| --- | --- | --- |
| Should do | Strong recommendations  based on strong evidence | Offer, should occur, provide, always do,  give, use, apply, (strongly) endorsed,  endorsed, must be considered, should be  considered, provide, promote and  facilitate, important to do, carried out,  primary choice, must be done, usually  appropriate |
| Could do | Weak recommendations  When a recommendation  could be considered | May include, recommend, practitioner  might, suggest, may be used, advice,  give, may be considered, conditional  recommendation, evaluate, can be  evaluated, can be tried, may be  appropriate, suggested, should be  carefully considered |
| Do not do | When a recommendation  should not be offered: recommendation against | Should refrain from, do not routinely  offer, not appropriate, should not, do not  give, do not use, do not, not indicated, do  not start |
| Uncertain | Inconclusive  recommendation: direction and strength of recommendation unclear | We are unable to recommend for or  against’, inconclusive evidence,  uncertain, no basis for recommending,  Insufficient evidence to make a  recommendation for or against |

# **Supplementary File 2. eResults**

## **Word text consistency of ChatGPT answers**

### **Table S1.** Consistency of ChatGPT 3.5 answers*

|  | CQ1_trial1.docx | CQ1_trial2.docx | CQ1_trial3.docx |
| --- | --- | --- | --- |
| CQ1_trial1.docx | N/A | 53% | 39% |
| CQ1_trial2.docx | 60% | N/A | 47% |
| CQ1_trial3.docx | 33% | 37% | N/A |
|  | CQ2_trial1.docx | CQ2_trial2.docx | CQ2_trial3.docx |
| CQ2_trial1.docx | N/A | 56% | 53% |
| CQ2_trial2.docx | 53% | N/A | 84% |
| CQ2_trial3.docx | 50% | 84% | N/A |
|  | CQ3_trial1.docx | CQ3_trial2.docx | CQ3_trial3.docx |
| CQ3_trial1.docx | N/A | 51% | 53% |
| CQ3_trial2.docx | 38% | N/A | 57% |
| CQ3_trial3.docx | 42% | 59% | N/A |
|  | CQ4_trial1.docx | CQ4_trial2.docx | CQ4_trial3.docx |
| CQ4_trial1.docx | N/A | 43% | 46% |
| CQ4_trial2.docx | 42% | N/A | 50% |
| CQ4_trial3.docx | 42% | 46% | N/A |
|  | CQ5_trial1.docx | CQ5_trial2.docx | CQ5_trial3.docx |
| CQ5_trial1.docx | N/A | 34% | 29% |
| CQ5_trial2.docx | 32% | N/A | 33% |
| CQ5_trial3.docx | 28% | 34% | N/A |
|  | CQ6_trial1.docx | CQ6_trial2.docx | CQ6_trial3.docx |
| CQ6_trial1.docx | N/A | 32% | 50% |
| CQ6_trial2.docx | 34% | N/A | 45% |
| CQ6_trial3.docx | 49% | 43% | N/A |
|  | CQ7_trial1.docx | CQ7_trial2.docx | CQ7_trial3.docx |
| CQ7_trial1.docx | N/A | 35% | 35% |
| CQ7_trial2.docx | 30% | N/A | 37% |
| CQ7_trial3.docx | 37% | 41% | N/A |
|  | CQ8_trial1.docx | CQ8_trial2.docx | CQ8_trial3.docx |
| CQ8_trial1.docx | N/A | 59% | 69% |
| CQ8_trial2.docx | 51% | N/A | 52% |
| CQ8_trial3.docx | 64% | 57% | N/A |
|  | CQ9_trial1.docx | CQ9_trial2.docx | CQ9_trial3.docx |
| CQ9_trial1..docx | N/A | 77% | 85% |
| CQ9_trial2.docx | 76% | N/A | 73% |
| CQ9_trial3.docx | 81% | 71% | N/A |

Legend: CQ, Clinical Question.

* Data from Gianola S, Bargeri S, Castellini G, et al. Performance of ChatGPT Compared to Clinical Practice Guidelines in Making Informed Decisions for Lumbosacral Radicular Pain: A Cross-sectional Study. *J Orthop Sports Phys Ther*. 2024;54(3):1-7. doi:10.2519/jospt.2024.12151

### **Table S2.** Consistency of ChatGPT 4o answers

|  | CQ1 TRIAL1.docx | CQ1 TRIAL2.docx | CQ1 TRIAL3.docx |
| --- | --- | --- | --- |
| CQ1 TRIAL1.docx | N/A | 28% | 34% |
| CQ1 TRIAL2.docx | 26% | N/A | 21% |
| CQ1 TRIAL3.docx | 36% | 23% | N/A |
|  | CQ2 TRIAL1.docx | CQ2 TRIAL2.docx | CQ2 TRIAL3.docx |
| CQ2 TRIAL1.docx | N/A | 23% | 29% |
| CQ2 TRIAL2.docx | 22% | N/A | 18% |
| CQ2 TRIAL3.docx | 24% | 17% | N/A |
|  | CQ3 TRIAL1.docx | CQ3 TRIAL2.docx | CQ3 TRIAL3.docx |
| CQ3 TRIAL1.docx | N/A | 38% | 24% |
| CQ3 TRIAL2.docx | 34% | N/A | 22% |
| CQ3 TRIAL3.docx | 18% | 20% | N/A |
|  | CQ4 TRIAL1.docx | CQ4 TRIAL2.docx | CQ4 TRIAL3.docx |
| CQ4 TRIAL1.docx | N/A | 31% | 28% |
| CQ4 TRIAL2.docx | 26% | N/A | 28% |
| CQ4 TRIAL3.docx | 27% | 36% | N/A |
|  | CQ5 TRIAL1.docx | CQ5 TRIAL2.docx | CQ5 TRIAL3.docx |
| CQ5 TRIAL1.docx | N/A | 37% | 26% |
| CQ5 TRIAL2.docx | 33% | N/A | 28% |
| CQ5 TRIAL3.docx | 20% | 25% | N/A |
|  | CQ6 TRIAL1.docx | CQ6 TRIAL2.docx | CQ6 TRIAL3.docx |
| CQ6 TRIAL1.docx | N/A | 36% | 30% |
| CQ6 TRIAL2.docx | 35% | N/A | 31% |
| CQ6 TRIAL3.docx | 27% | 28% | N/A |
|  | CQ7 TRIAL1.docx | CQ7 TRIAL2.docx | CQ7 TRIAL3.docx |
| CQ7 TRIAL1.docx | N/A | 0% | 20% |
| CQ7 TRIAL2.docx | 0% | N/A | 0% |
| CQ7 TRIAL3.docx | 17% | 0% | N/A |
|  | CQ8 TRIAL1.docx | CQ8 TRIAL2.docx | CQ8 TRIAL3.docx |
| CQ8 TRIAL1.docx | N/A | 26% | 28% |
| CQ8 TRIAL2.docx | 24% | N/A | 33% |
| CQ8 TRIAL3.docx | 23% | 31% | N/A |
|  | CQ9 TRIAL1.docx | CQ9 TRIAL2.docx | CQ9 TRIAL3.docx |
| CQ9 TRIAL1.docx | N/A | 26% | 33% |
| CQ9 TRIAL2.docx | 22% | N/A | 31% |
| CQ9 TRIAL3.docx | 32% | 32% | N/A |

Legend: CQ, Clinical Question

### **Table S3.** Consistency of Cloud answers

|  | CQ1 TRIAL1.docx | CQ1 TRIAL2.docx | CQ1 TRIAL3.docx |
| --- | --- | --- | --- |
| CQ1 TRIAL1.docx | N/A | 27% | 25% |
| CQ1 TRIAL2.docx | 29% | N/A | 40% |
| CQ1 TRIAL3.docx | 28% | 41% | N/A |
|  | CQ2 TRIAL1.docx | CQ2 TRIAL2.docx | CQ2 TRIAL3.docx |
| CQ2 TRIAL1.docx | N/A | 51% | 35% |
| CQ2 TRIAL2.docx | 48% | N/A | 41% |
| CQ2 TRIAL3.docx | 30% | 34% | N/A |
|  | CQ3 TRIAL1.docx | CQ3 TRIAL2.docx | CQ3 TRIAL3.docx |
| CQ3 TRIAL1.docx | N/A | 31% | 41% |
| CQ3 TRIAL2.docx | 31% | N/A | 28% |
| CQ3 TRIAL3.docx | 46% | 33% | N/A |
|  | CQ4 TRIAL1.docx | CQ4 TRIAL2.docx | CQ4 TRIAL3.docx |
| CQ4 TRIAL1.docx | N/A | 46% | 29% |
| CQ4 TRIAL2.docx | 44% | N/A | 20% |
| CQ4 TRIAL3.docx | 23% | 20% | N/A |
|  | CQ5 TRIAL1.docx | CQ5 TRIAL2.docx | CQ5 TRIAL3.docx |
| CQ5 TRIAL1.docx | N/A | 23% | 25% |
| CQ5 TRIAL2.docx | 25% | N/A | 32% |
| CQ5 TRIAL3.docx | 28% | 34% | N/A |
|  | CQ6 TRIAL1.docx | CQ6 TRIAL2.docx | CQ6 TRIAL3.docx |
| CQ6 TRIAL1.docx | N/A | 39% | 38% |
| CQ6 TRIAL2.docx | 43% | N/A | 36% |
| CQ6 TRIAL3.docx | 43% | 38% | N/A |
|  | CQ7 TRIAL1.docx | CQ7 TRIAL2.docx | CQ7 TRIAL3.docx |
| CQ7 TRIAL1.docx | N/A | 18% | 28% |
| CQ7 TRIAL2.docx | 20% | N/A | 29% |
| CQ7 TRIAL3.docx | 30% | 30% | N/A |
|  | CQ8 TRIAL1.docx | CQ8 TRIAL2.docx | CQ8 TRIAL3.docx |
| CQ8 TRIAL1.docx | N/A | 23% | 24% |
| CQ8 TRIAL2.docx | 30% | N/A | 30% |
| CQ8 TRIAL3.docx | 30% | 29% | N/A |
|  | CQ9 TRIAL1.docx | CQ9 TRIAL2.docx | CQ9 TRIAL3.docx |
| CQ9 TRIAL1.docx | N/A | 41% | 50% |
| CQ9 TRIAL2.docx | 43% | N/A | 42% |
| CQ9 TRIAL3.docx | 47% | 38% | N/A |

Legend: CQ, Clinical Question

### **Table S4.** Consistency of Microsoft Copilot answers

|  | CQ1 TRIAL 1.docx | CQ1 TRIAL 2.docx | CQ1 TRIAL 3.docx |
| --- | --- | --- | --- |
| CQ1 TRIAL 1.docx | N/A | 44% | 68% |
| CQ1 TRIAL 2.docx | 57% | N/A | 44% |
| CQ1 TRIAL 3.docx | 71% | 35% | N/A |
|  | CQ2 TRIAL 1.docx | CQ2 TRIAL 2.docx | CQ2 TRIAL 3.docx |
| CQ2 TRIAL 1.docx | N/A | 57% | 63% |
| CQ2 TRIAL 2.docx | 70% | N/A | 65% |
| CQ2 TRIAL 3.docx | 83% | 69% | N/A |
|  | CQ3 TRIAL 1.docx | CQ3 TRIAL 2.docx | CQ3 TRIAL 3.docx |
| CQ3 TRIAL 1.docx | N/A | 83% | 94% |
| CQ3 TRIAL 2.docx | 70% | N/A | 70% |
| CQ3 TRIAL 3.docx | 77% | 68% | N/A |
|  | CQ4 TRIAL 1.docx | CQ4 TRIAL 2.docx | CQ4 TRIAL 3.docx |
| CQ4 TRIAL 1.docx | N/A | 87% | 83% |
| CQ4 TRIAL 2.docx | 96% | N/A | 92% |
| CQ4 TRIAL 3.docx | 87% | 88% | N/A |
|  | CQ5 TRIAL 1.docx | CQ5 TRIAL 2.docx | CQ5 TRIAL 3.docx |
| CQ5 TRIAL 1.docx | N/A | 94% | 81% |
| CQ5 TRIAL 2.docx | 98% | N/A | 85% |
| CQ5 TRIAL 3.docx | 78% | 78% | N/A |
|  | CQ6 TRIAL 1.docx | CQ6 TRIAL 2.docx | CQ6 TRIAL 3.docx |
| CQ6 TRIAL 1.docx | N/A | 33% | 64% |
| CQ6 TRIAL 2.docx | 40% | N/A | 36% |
| CQ6 TRIAL 3.docx | 80% | 39% | N/A |
|  | CQ7 TRIAL 1.docx | CQ7 TRIAL 2.docx | CQ7 TRIAL 3.docx |
| CQ7 TRIAL 1.docx | N/A | 69% | 88% |
| CQ7 TRIAL 2.docx | 63% | N/A | 63% |
| CQ7 TRIAL 3.docx | 88% | 69% | N/A |
|  | CQ8 TRIAL 1.docx | CQ8 TRIAL 2.docx | CQ8 TRIAL 3.docx |
| CQ8 TRIAL 1.docx | N/A | 74% | 46% |
| CQ8 TRIAL 2.docx | 61% | N/A | 34% |
| CQ8 TRIAL 3.docx | 42% | 38% | N/A |
|  | CQ9 TRIAL 1.docx | CQ9 TRIAL 2.docx | CQ9 TRIAL 3.docx |
| CQ9 TRIAL 1.docx | N/A | 58% | 54% |
| CQ9 TRIAL 2.docx | 54% | N/A | 43% |
| CQ9 TRIAL 3.docx | 52% | 44% | N/A |

Legend: CQ, Clinical Question

### **Table S5.** Consistency of Gemini answers

|  | CQ1 TRIAL1.docx | CQ1 TRIAL2.docx | CQ1 TRIAL3.docx |
| --- | --- | --- | --- |
| CQ1 TRIAL1.docx | N/A | 30% | 28% |
| CQ1 TRIAL2.docx | 25% | N/A | 20% |
| CQ1 TRIAL3.docx | 29% | 22% | N/A |
|  | CQ2 TRIAL1.docx | CQ2 TRIAL2.docx | CQ2 TRIAL3.docx |
| CQ2 TRIAL1.docx | N/A | 27% | 29% |
| CQ2 TRIAL2.docx | 30% | N/A | 23% |
| CQ2 TRIAL3.docx | 30% | 21% | N/A |
|  | CQ3 TRIAL1.docx | CQ3 TRIAL2.docx | CQ3 TRIAL3.docx |
| CQ3 TRIAL1.docx | N/A | 31% | 32% |
| CQ3 TRIAL2.docx | 22% | N/A | 28% |
| CQ3 TRIAL3.docx | 32% | 40% | N/A |
|  | CQ4 TRIAL1.docx | CQ4 TRIAL2.docx | CQ4 TRIAL3.docx |
| CQ4 TRIAL1.docx | N/A | 23% | 24% |
| CQ4 TRIAL2.docx | 22% | N/A | 27% |
| CQ4 TRIAL3.docx | 21% | 26% | N/A |
|  | CQ5 TRIAL1.docx | CQ5 TRIAL2.docx | CQ5 TRIAL3.docx |
| CQ5 TRIAL1.docx | N/A | 23% | 11% |
| CQ5 TRIAL2.docx | 19% | N/A | 18% |
| CQ5 TRIAL3.docx | 12% | 22% | N/A |
|  | CQ6 TRIAL1.docx | CQ6 TRIAL2.docx | CQ6 TRIAL3.docx |
| CQ6 TRIAL1.docx | N/A | 24% | 35% |
| CQ6 TRIAL2.docx | 17% | N/A | 17% |
| CQ6 TRIAL3.docx | 33% | 18% | N/A |
|  | CQ7 TRIAL1.docx | CQ7 TRIAL2.docx | CQ7 TRIAL3.docx |
| CQ7 TRIAL1.docx | N/A | 24% | 33% |
| CQ7 TRIAL2.docx | 30% | N/A | 34% |
| CQ7 TRIAL3.docx | 31% | 26% | N/A |
|  | CQ8 TRIAL1.docx | CQ8 TRIAL2.docx | CQ8 TRIAL3.docx |
| CQ8 TRIAL1.docx | N/A | 64% | 100% |
| CQ8 TRIAL2.docx | 54% | N/A | 54% |
| CQ8 TRIAL3.docx | 100% | 64% | N/A |
|  | CQ9 TRIAL1.docx | CQ9 TRIAL2.docx | CQ9 TRIAL3.docx |
| CQ9 TRIAL1.docx | N/A | 32% | 33% |
| CQ9 TRIAL2.docx | 38% | N/A | 72% |
| CQ9 TRIAL3.docx | 38% | 70% | N/A |

Legend: CQ, Clinical Question

### **Table S6.** Consistency of Perplexity answers

|  | CQ1 trial 1.docx | CQ1 trial 2.docx | CQ1 trial 3.docx |
| --- | --- | --- | --- |
| CQ1 trial 1.docx | N/A | 62% | 71% |
| CQ1 trial 2.docx | 54% | N/A | 63% |
| CQ1 trial 3.docx | 56% | 58% | N/A |
|  | CQ2 trial 1.docx | CQ2 trial 2.docx | CQ2 trial 3.docx |
| CQ2 trial 1.docx | N/A | 39% | 49% |
| CQ2 trial 2.docx | 28% | N/A | 32% |
| CQ2 trial 3.docx | 36% | 35% | N/A |
|  | CQ3 trial 1.docx | CQ3 trial 2.docx | CQ3 trial 3.docx |
| CQ3 trial 1.docx | N/A | 53% | 57% |
| CQ3 trial 2.docx | 66% | N/A | 96% |
| CQ3 trial 3.docx | 68% | 93% | N/A |
|  | CQ4 trial 1.docx | CQ4 trial 2.docx | CQ4 trial 3.docx |
| CQ4 trial 1.docx | N/A | 71% | 71% |
| CQ4 trial 2.docx | 60% | N/A | 78% |
| CQ4 trial 3.docx | 62% | 76% | N/A |
|  | CQ5 trial 1.docx | CQ5 trial 2.docx | CQ5 trial 3.docx |
| CQ5 trial 1.docx | N/A | 41% | 65% |
| CQ5 trial 2.docx | 45% | N/A | 27% |
| CQ5 trial 3.docx | 71% | 32% | N/A |
|  | CQ6 trial 1.docx | CQ6 trial 2.docx | CQ6 trial 3.docx |
| CQ6 trial 1.docx | N/A | 64% | 63% |
| CQ6 trial 2.docx | 85% | N/A | 85% |
| CQ6 trial 3.docx | 64% | 64% | N/A |
|  | CQ7 trial 1.docx | CQ7 trial 2.docx | CQ7 trial 3.docx |
| CQ7 trial 1.docx | N/A | 63% | 71% |
| CQ7 trial 2.docx | 58% | N/A | 92% |
| CQ7 trial 3.docx | 67% | 94% | N/A |
|  | CQ8 trial 1.docx | CQ8 trial 2.docx | CQ8 trial 3.docx |
| CQ8 trial 1.docx | N/A | 49% | 46% |
| CQ8 trial 2.docx | 41% | N/A | 45% |
| CQ8 trial 3.docx | 43% | 53% | N/A |
|  | CQ9 trial 1.docx | CQ9 trial 2.docx | CQ9 trial 3.docx |
| CQ9 trial 1.docx | N/A | 64% | 65% |
| CQ9 trial 2.docx | 33% | N/A | 34% |
| CQ9 trial 3.docx | 35% | 38% | N/A |

Legend: CQ, Clinical Question

.
